# Synthetic microbiology: from analogy to methodology

**DOI:** 10.1111/1751-7915.12786

**Published:** 2017-07-26

**Authors:** Víctor de Lorenzo

**Affiliations:** ^1^ Systems Biology Program Centro Nacional de Biotecnología (CNB‐CSIC) 28049 Cantoblanco‐Madrid Spain

From the onset of genetic engineering in the mid‐70 of the last century, microbiologists have entertained programming environmental bacteria for the sake of industrial and ultimately global sustainability. The earlier agenda included not only using microorganisms as catalysts of reactions and processes alternative to those adopted by the chemical industry, but also the development of microbial agents for extensive release either to improve crop yields or for *in situ* bioremediation of pollutants (Lindow *et al*., 1989). Alas, besides early concerns on safety and the ensuing regulatory limitations about liberation of GMOs,^1^ the fact is that the scientists of the time failed to deliver much of the environmental and agricultural applications envisioned then for recombinant DNA technology. Besides encountering many unexpected microbial ecology challenges along the way (which, however, subsequently fostered new perspectives in the field that reaches us to this day), the reality of what was called at the time *genetic engineering* (GE) had little to do with authentic *engineering*. The latter is characterized by serious metrology, standards, definition of systems’ components and boundaries, transfer functions, relational logic, modularity, reusability, robust modelling and many other features that have been traditionally alien to Life Sciences research (de Lorenzo and Schmidt, 2017). In contrast, what we have generally called genetic engineering would be better described as *genetic bricolage* or *DNA tinkering*, in which genes (most often one or few at a time) are minimally manipulated and passed from their original host to another for enhancing or modifying their activity with a desired purpose. The authentic engineering aspect of such endeavours, which have dominated microbial biotechnology for decades, is close to zero: it has been just an inspiring analogy, a flamboyant metaphor – but no *bona fide* engineering. Although the success stories of such a *trial‐and‐error Biotechnology* have been many, the time has come for a major change in the way we try to modify – and ultimately build from first principles – biological systems, in particular microorganisms.

The broker of this new phase is the discipline we call Synthetic Biology (SynBio) which, capitalizing on the quantitative spirit of Systems Biology, looks at biological objects (from metabolites and proteins to whole cells) through the eyes of real (not metaphoric) engineering (Andrianantoandro *et al*., 2006). This involves a new interpretative frame of living entities that is compatible with, but different from, the standard evolutionary and molecular biology views that have prevailed in Biology since elucidation of the structure of DNA. While the essence of Molecular Biology relies on the so‐called central dogma (DNA → RNA → Proteins), SynBio leaves aside the evolutionary origin of biological systems and addresses instead the compositional and relational logic that makes biological systems work the way they do.^2^ This change of perspective implies adoption of a different abstraction hierarchy, namely Parts → Devices → Systems. In this way, SynBio allows (i) understanding the functioning of live systems out of its physicochemical and spatial casting, (ii) modifying and combining rationally existing properties for enhancing or crafting new ones, and (iii) creating altogether new‐to‐nature biological activities and materials.

One can immediately appreciate that the ongoing happy encounter between Life Sciences and Engineering that is the essence of SynBio can have the same profound impact in our relation with living systems as Physics had at the outset of Molecular Biology. In reality – and despite the many gaps in knowledge and multiple thus far unsolved tasks – SynBio puts in our hands an unprecedented power to revisit many of the earlier promises of GE, including those that dealt with environmental sustainability. Moreover, SynBio allows the tackling of new challenges, the scale and complexity of which previously ruled out traditional genetic engineering as a technological choice to meet them.

By enabling the design of microorganisms *à la carte,* one can envision the increase in the range of compounds (from high added‐value molecules to bulk chemicals) that can be produced in an environmentally friendly fashion to meet a large number of human necessities – from drugs to new materials – and thus decrease undesirable emissions (Lee *et al*., 2012). By the same token, we can revisit also the design of agents for detection (i.e. biosensing; Merulla *et al*., 2013) and bioremediation of thus far intractable chemical pollutants. In the event that some enzymatic activities to this end are not naturally available, SynBio proposes to invent them from scratch (Walther *et al*., 2017), or to evolve them from focused diversification of DNA sequences. Note that while the earlier agenda of traditional GE‐based bioremediation schemes focused on getting rid of the toxic chemicals at stake, SynBio‐enabled metabolic engineering allows valorization of waste, a far more appealing process than its mere destruction (Wierckx *et al*., 2015). And SynBio may additionally allow us to slow down or even revert some of the more pressing global environmental problems, for example global warming due to greenhouse gas emissions, plastic pollution of oceans and desertification of land (de Lorenzo *et al*., 2016). Also, bacteria (including complex root‐associated microbial communities) could be remodelled for the sake of a better agronomical productivity. And, last but not least, our gut microbiome will also be the subject of intensive research as the target of therapeutic bacteria that both sense and respond to specific health conditions (de la Fuente‐Nunez *et al*., 2017). Along this line, phage‐based and CRISPR‐based therapies to treat bacterial infections not amenable to standard antibiotics are emerging as a powerful strategy to combat obstinate pathogens and to selectively kill virulent or antibiotic‐resistant subpopulations of a given species (Barbu *et al*., 2016).

But microorganisms are not alone in the Earth ecosystems. SynBio can do more to meet the large‐scale SDGs dealing with combating desertification and halt biodiversity loss. Industrial and agricultural expansion have often resulted in the disappearance of the habitats of a large number of animals, the displacement of indigenous vegetation and the endangering of the multiscale biological diversity of the sites afflicted – from bacteria to plants to large mammals. SynBio opens new perspectives to Conservation Biology (Redford *et al*., 2014) that will safeguard and enrich, rather than reduce the entire landscape of the biological realm as we know it now. The ease of massive DNA sequence, along with CRISPR‐based gene editing methods, will allow not only to keep a virtually complete inventory of every species but also to go back down the evolutionary tree and bring back to life variants that were long extinguished – including, e.g., ancestral enzymes and microorganisms – and to then decide strategies for re‐implantation of key species in target ecosystems. Some low‐hanging fruits of these approaches include the recovery of emblematic species that are currently on the verge of disappearance^3^ and the ongoing efforts to resuscitate (i.e. de‐extinction) animals, plants and enzymes recently lost (Friese and Marris, 2014). But sustainable survival of fragile species (other than having them in zoos, aquariums and reservations) has to go hand‐in‐hand with the recovery of their corresponding ecosystems, often sustained ultimately by microorganisms. This is an endeavour that is tractable through biologically based environmental interventions, e.g. aimed at rehabilitation of dry/waste lands or de‐eutrophication of water. Yet, it should be noted that much of the current loss of biodiversity stems from deliberate human decisions on the use of land and not from any inevitable biological process. While the Biotechnology of the future cannot by itself solve what is essentially a global political problem, it can provide models, methods and agents that enable the re‐establishment of sustainable ecosystems. One promising approach is the ongoing design of biological constructs able to bring about new physicochemical conditions on which fresh trophic chains can build spontaneously (Sole, 2015; Sole *et al*., 2015). In reality, tackling many such large‐scale biodiversity challenges, which are also related to climate change and excess of greenhouse gases emissions, is not so much a technical problem, but a matter of global governance, which SynBio can support but by no means replace.

In the meantime, laboratories and Biotechnology companies will keep on producing increasing numbers of engineered biological objects for specific purposes, including, e.g., live microorganisms. And there is a legitimate concern about the uncertain effects of such new‐to‐nature items in the extant ecosystems. This issue has been on top of the table since the 1975 Asilomar Conference and still remains a matter of public preoccupation. Earlier approaches to biological and genetic containment of engineered agents were based on conditional survival systems that ensured that given constructs could not escape predetermined time‐and‐space restrictions (de Lorenzo, 2010). In contrast, modern‐day approaches pursue methods for *absolute certainty of containment* in which the genetic material of a GM organism cannot be *understood* at all by any naturally occurring host (Schmidt and de Lorenzo, 2012). Attempts to this end include recoding the entire genome of the GMO at stake, making it dependent on a xenobiotic compound, altering/expanding the genetic code or using an alternative nucleic acid structure to isolate the biological information. In this way, there is also a sort of *linguistic containment* that should stop the flow of any human‐designed genetic information to/from ordinary biological hosts (Schmidt and de Lorenzo, 2016). Once more, the technology to tackle the problem is already there, but how much gene transfer is acceptable in the context of regulations and governance has to be decided with participation of other stakeholders. Questions such as whether GM biosystems of the future must be entirely orthogonal (i.e. independent) of natural counterparts for avoidance of artificial *genetic pollution* of the natural world still require a considerable debate and more evidence on the actual limits of the firewalls entertained thus far.

## Conflict of interest

None declared.

## Notes

1 Genetically Manipulated Organism

2 https://www.youtube.com/watch?v=sQl2TYTqSDQ

3 https://www.scientificamerican.com/article/lost-species-revived-from-dna-and-restored-to-nature/
